# In vitro comparison of CD20xCD3 bispecific antibodies against diffuse large B‐cell lymphoma (DLBCL) cell lines with different levels of expression of CD20

**DOI:** 10.1111/bjh.20033

**Published:** 2025-03-03

**Authors:** Joshua S. Bray, Gethin R. Thomas, Victoria M. Smith, Adam Wright, Sandrine Jayne, Martin J. S. Dyer, Harriet S. Walter

**Affiliations:** ^1^ The Ernest and Helen Scott Haematological Research Institute, Leicester Cancer Research Centre University of Leicester Leicester UK; ^2^ Respiratory Sciences University of Leicester, Glenfield Hospital Leicester UK; ^3^ Department of Oncology University Hospitals Leicester Leicester UK

**Keywords:** antibody therapy, bispecific antibodies, CD20, lymphoid malignancies

## Abstract

Although CD20xCD3 bispecific antibodies (BsAbs) have demonstrated transformational activity in diffuse large B‐cell lymphoma (DLBCL), some patients fail to respond and others relapse. To begin to explore possible limitations, we compared the in vitro activity of four CD20xCD3 biosimilar BsAbs against four DLBCL cell lines with CD20 expression ranging over a 100‐fold. All four biosimilar BsAbs demonstrated superior in vitro activity to rituximab, with biosimilar glofitamab consistently being the most potent. Moreover, biosimilar glofitamab and odronextamab retained significant activity in the presence of low‐level CD20 expression. Finally, one DLBCL cell line exhibited intrinsic resistance to all four CD20xCD3 BsAbs despite inducing marked T‐cell and NK‐cell activation.

## INTRODUCTION

Until recently, most patients with relapsed/refractory (R/R) DLBCL died rapidly from uncontrolled disease.^1^ T‐cell engaging therapies including chimeric antigen receptor (CAR‐T) T‐cells and CD20xCD3 BsAbs, both of which can induce durable remissions, have transformed this clinical situation.^2^, ^3^ Nevertheless, not all patients respond, whilst others relapse. Some biological variables of DLBCL associated with poor responses to CAR‐T cells have been determined.^4^, ^5^ Importantly, in the context of both myeloma and B‐cell precursor acute lymphoblastic leukemia (ALL), very low levels of target antigen (CD19 and CD20) expression may not be a barrier to CAR‐T efficacy.^6^, ^7^ Malignant B cells expressing less than 1000 molecules of either CD19 or CD20, below the levels detectable by immunohistochemistry and flow cytometry, can be successfully targeted in vitro and in vivo by CAR‐T cells.^8^ Whether similarly low level CD20 expression undetectable by immunohistochemistry is permissive for specific CD20xCD3 BsAbs responses, which form a different immune synapse with malignant B cells from CAR‐T cells, remains unknown.^9^, ^10^, ^11^ Using a panel of four biosimilar CD20xCD3 BsAbs, we have compared the efficacy against DLBCL cell lines with defined levels of expression of CD20.

## METHODS

Cell lines and biosimilar monospecific mAbs and BsAbs used in this study are described in the Supporting Information and Table S1. CD20 protein expression was quantified using the Dako QiFi kit (Agilent Dako). BsAb activity was assessed using a flow cytometry‐based cytotoxicity assay as previously described with modifications.^12^ Briefly, target B cell lines with different levels of CD20 expression were treated with a range of concentrations of biosimilar BsAb (0.01–10 000 pM) or monospecific antibody biosimilar (0.1–100 000 pM) (Proteogenix) and with healthy volunteer peripheral blood mononuclear cells (PBMCs) as effectors for 24 h. Effector PBMCs were depleted of B cells using CD20 microbeads (Miltenyi Biotec) prior to co‐culture. Target B cell depletion (BCD) was calculated by comparing live target cell counts with a no drug control. Effector T and natural killer (NK) cell activation were determined by the expression of CD69 (early activation), CD25 (late activation) and CD107a (degranulation). Absolute EC_50_ values were estimated using a four‐parameter log‐logistic regression model.

## RESULTS

**FIGURE 1:**
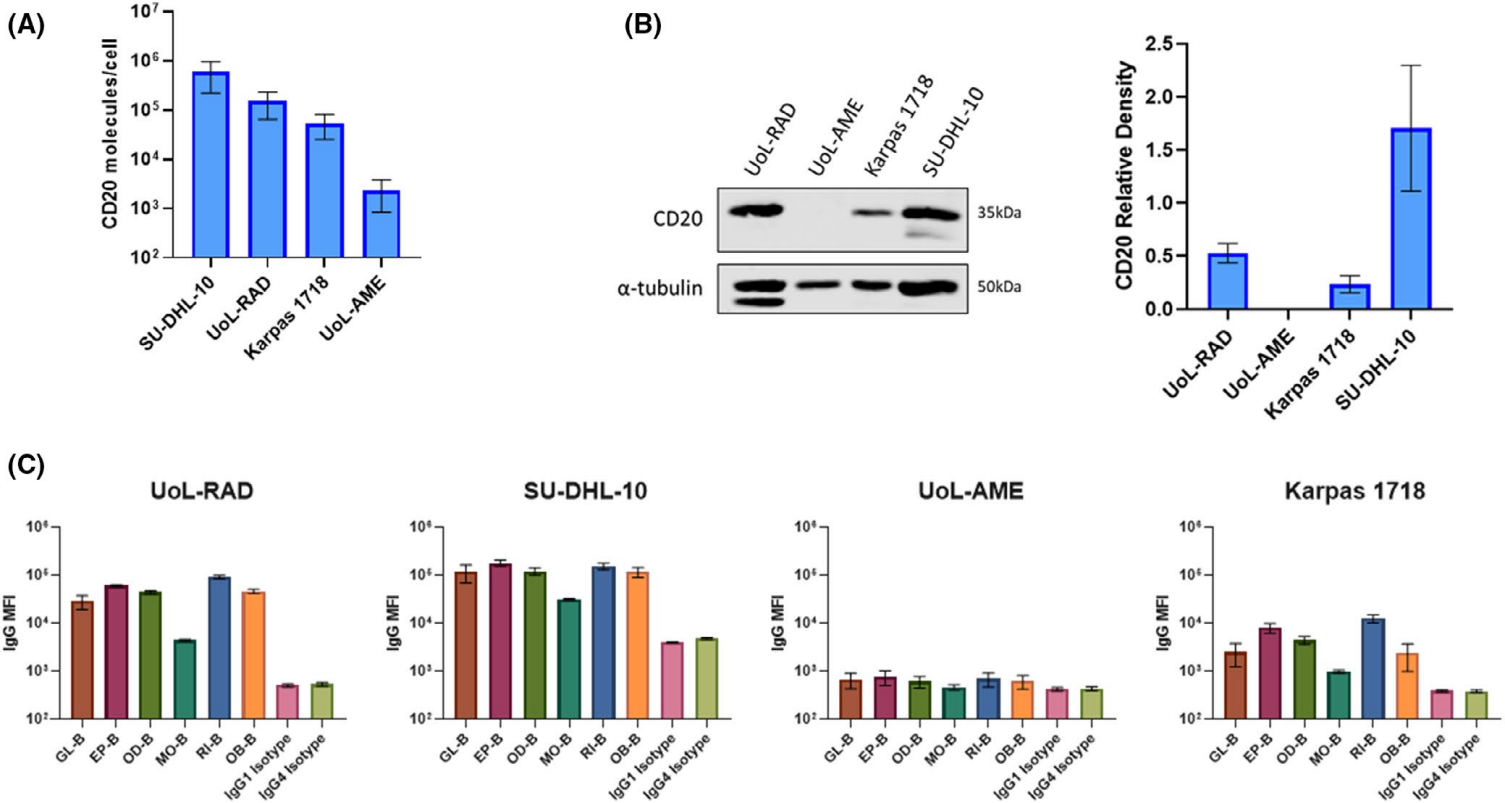
Variable CD20 expression across DLBCL cell lines. (A) QiFi quantification of surface CD20 (mean ± SD) in DLBCL cell lines. *N* = 3–5. (B) CD20 expression determined by immunoblot in DLBCL whole cell lysates, normalised to alpha‐tubulin (mean ± SD). *N* = 4. (C) Median fluorescence intensity (mean ± SD) of surface‐bound CD20 antibody biosimilar. Cells were incubated with 10 nM CD20 antibody biosimilar for 1 h at 4°C, then stained with anti‐human IgG‐APC. *N* = 3.

**FIGURE 2:**
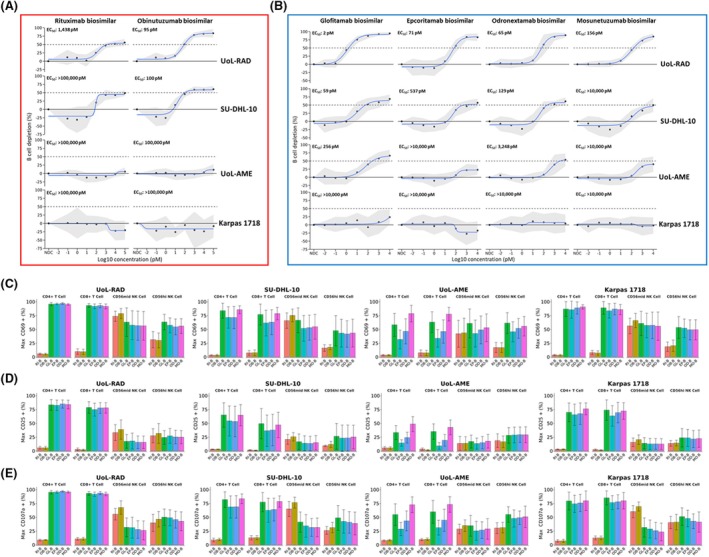
In vitro efficacy of CD20 antibody biosimilars against DLBCL cell lines. DLBCL cells were treated with CD20 monospecific mAb or CD20xCD3 BsAb for 24 h using healthy volunteer PBMCs at an effector: target ratio of 6:1. *N* = 6–12. (A) B cell depletion (mean ± SD) efficacy of monospecific CD20 mAbs. (B) B cell depletion (mean ± SD) efficacy of CD20xCD3 BsAbs. (C–E) Maximum expression (mean ± SD) of (C) CD69, (D) CD25 and (E) CD107a in effector cell subsets. EP‐B, epcoritamab biosimilar; GL‐B, glofitamab biosimilar; MO‐B, mosunetuzumab biosimilar; OB‐B, obinutuzumab biosimilar; OD‐B, odronextamab biosimilar; RI‐B, rituximab biosimilar.

The four DLBCL cell lines expressed different levels of cell surface CD20 ranging from approximately 2000 to 200 000 molecules/cell detected by flow cytometry using the 2H7 mAb (Figure 1A). CD20 expression was strongly detectable by immunoblot in UoL‐RAD and SU‐DHL‐10, more weakly in Karpas 1718, but was undetectable in UoL‐AME (Figure 1B). Cell surface staining by all six biosimilar CD20 antibodies is summarized in Figure 1C. There were some differences in staining intensity between constructs, most notably mosunetuzumab, with an MFI which was up to 14‐fold lower compared to the other CD20 antibodies. Similar patterns of staining were observed in UoL‐RAD and SU‐DHL‐10; low staining intensity was observed in UoL‐AME with all antibodies. B‐cell depletion (BCD) showed variable results according to antibody and cell line (Figure 2). For the monospecific CD20 mAbs, rituximab and obinutuzumab, both the UoL‐RAD and SU‐DHL‐10 cell lines were sensitive, with obinutuzumab producing slightly higher maximum BCD (UoL‐RAD BCD 85.0 ± standard deviation [SD] 10.6, EC_50_ 95 pM; SU‐DHL‐10 BCD 64.3 ± 6.93, EC_50_ 100 pM) than rituximab (UoL‐RAD BCD 56.7 ± SD 9.2, EC_50_ 1438 pM; SU‐DHL‐10 BCD 51.3 ± 9.5, EC_50_ > 100 000 pM) (Figure 2A). In these cell lines, rituximab and obinutuzumab induced dose‐dependent NK cell activation and degranulation with no significant T cell activation as anticipated. In contrast, neither Karpas 1718 nor UoL‐AME showed significant BCD with either monospecific mAb (Karpas 1718 obinutuzumab BCD 14.0 ± SD 15.6, rituximab BCD 22.0 ± 23.4; UoL‐AME obinutuzumab BCD 13.0 ± SD 14.4, rituximab BCD 10.4 ± 8.1). There was minimal engagement of NK cells by rituximab or obinutuzumab against UoL‐AME, while against Karpas 1718 there was significant NK cell activation. Further subclassification of NK cells into CD56mid and CD56hi populations revealed that monospecific antibodies induced greater activation and degranulation in CD56mid cells compared to CD56hi cells.

For the CD20xCD3 BsAbs, both rituximab‐sensitive cell lines were sensitive to all four BsAbs, albeit to differing degrees. All four CD20xCD3 BsAbs displayed superior efficacy to rituximab, indicated by a higher maximal BCD and/or lower EC_50_ values; the glofitamab biosimilar demonstrated the highest maximal killing and was consistently the most potent (UoL‐RAD EC_50_ 2 pM; SU‐DHL‐10 EC_50_ 59 pM; UoL‐AME EC_50_ 256 pM; Karpas 1718 EC_50_ > 10 000 pM) (Figure 2B). All BsAb biosimilars engaged CD4+ and CD8+ T cells to similar degrees, indicated by dose‐dependent increases in CD69, CD25, and CD107a expression (Figure 2C–E).

UoL‐AME, despite its very low‐level expression of CD20, exhibited marked sensitivity to both glofitamab (EC_50_ 256 pM) and odronextamab (EC_50_ 3248 pM) but was only poorly sensitive to mosunetuzumab (EC_50_ > 10 000 pM) and resistant to epcoritamab (EC_50_ > 10 000 pM). With this cell line, the maximal T cell activity was much more variable between BsAbs, with mosunetuzumab showing the greatest effect.

In contrast, Karpas 1718 was the only model which was resistant to all four CD20xCD3 BsAbs, despite expressing 10^5^ molecules per cell of CD20 (compared to 10^3^ in UoL‐AME) and exhibiting marked engagement of both CD4+ and CD8+ populations.

Surprisingly, NK cell activation and degranulation were observed following BsAb exposure, particularly within the CD56hi subset. This phenomenon was observed across all cell lines.

## DISCUSSION

CD20xCD3 BsAbs have transformational single‐agent activity in R/R diffuse large B‐cell lymphoma (DLBCL) and are currently being assessed in front‐line therapy. However, several significant problems remain. First, the features that limit the clinical efficacy of BsAbs remain largely unknown. Second, there are significant differences in the design of the CD20xCD3 BsAbs; which of these BsAbs is superior awaits head‐to‐head clinical evaluation. However, to begin to address both problems, we have assessed the in vitro activity of four CD20xCD3 biosimilar BsAbs against DLBCL cell lines with levels of CD20 expression varying over a 100x range. Some preliminary conclusions may be drawn.

First, all BsAbs tested demonstrated superior in vitro activity to rituximab; these data align with clinical observations which show that CD20xCD3 BsAbs retain significant activity in patients who have failed rituximab‐based immunochemotherapy. Second, our data indicate that the glofitamab biosimilar had superior efficacy compared to other CD20xCD3 constructs, which may be due to its unique 2:1 (CD20:CD3) binding configuration. Preclinical studies of glofitamab have demonstrated that a 2:1 format confers a 40‐fold decrease in EC_50_ in comparison to other BsAbs, which is also reflected in our data.^12^ Whether this in vitro observation translates to improved clinical outcomes remains uncertain.

Third, our data indicate that the efficacy of CD20xCD3 BsAbs, like CAR‐T cells, may not be constrained by low levels of expression of CD20. Significant in vitro responses to glofitamab and odronextamab BsAbs were observed for UoL‐AME, despite its low CD20 expression. These data suggest that a relatively low number of binding events are required for effective T‐cell killing, which mirrors the antigen‐specific T‐cell response, where a mere three TCR–MHC interactions are sufficient for cytotoxicity.^7^ Patients with CD20‐negative DLBCL by immunohistochemistry (such as the patient from which the UoL‐AME cell line was derived) might therefore be considered for CD20xCD3 BsAb therapy. However, BsAb construct selection may be key where antigen expression is low or absent. Clinical data to date support a potential role for glofitamab and odronextamab in these settings.^9^, ^11^ Bystander effects via activation of NK cells may also play a role. Our data suggest that BsAbs preferentially activate CD56hi NK cells, which represent the dominant NK‐cell subset within the secondary lymphoid tissues.^13^ Both in vitro and in vivo studies with mosunetuzumab have demonstrated comparable activation of NK cells through cytokine release, with IL2 and IFNγ identified as the main players.^14^ Thus, it is possible that this CD56hi subset specifically may contribute to the efficacy of BsAbs in vivo, as they possess a high‐affinity IL‐2 receptor. A skewing of NK cells towards a CD56hi phenotype has additionally been reported both in Hodgkin lymphoma and multiple myeloma, with the latter study demonstrating that these cells retain proliferative and cytotoxic activity.^15^

Finally, Karpas 1718 was resistant to all CD20 antibodies tested, both monospecific and bispecific, despite significant CD20 expression and effector cell engagement, suggesting a tumour‐intrinsic mechanism of resistance, which may relate to the quality of the immune synapse, resistance to cytotoxic mediators including perforin, granzymes, TNFα, IFNγ, FasL and TRAIL, or alternatively impairment of CD20 downstream signalling. Further studies are needed to characterize the mechanism of resistance in this model.

## AUTHOR CONTRIBUTIONS

JSB performed research, analysed data and wrote the manuscript; GRT performed research and analysed data; VMS analysed data; AW analysed data; SJ, MJSD and HSW supervised research and wrote the manuscript; and all authors approved the final version.

## FUNDING INFORMATION

This work was supported by funds from the Scott Waudby Trust, Leicester Experimental Cancer Medicine Centre (ECMC) [ECMCQQR‐2022/100006] and the University of Leicester institutional MRC Impact Accelerator Account [MR/X502777/1]. JSB was supported through a University of Leicester Institute for Precision Health PhD studentship. The research was carried out at the National Institute for Health and Care Research (NIHR) Leicester Biomedical Research Centre (BRC).

We wish to acknowledge funding from the University of Leicester, MAARA (Midlands Asthma & Allergy Research Association) and the NIHR BRC Respiratory & Infection theme (University Hospitals of Leicester) towards the purchase and maintenance of the Attune NxT cytometer, utilized in this study.

## CONFLICT OF INTEREST STATEMENT

The authors have no relevant conflicts of interest to disclose.

## ETHICS STATEMENT

This study was approved by a research ethics committee and sponsored by the University of Leicester (2024‐0243‐476).

## PATIENT CONSENT STATEMENT

Samples were obtained after written informed consent.

## PERMISSION TO REPRODUCE MATERIAL FROM OTHER SOURCES

No material from other sources is included.

## Supporting information


Data S1.

Table S1.

## Data Availability

Additional data that support the findings of this study are available from the corresponding author upon reasonable request.
