# Supplementary material for: In vitro comparison of CD20xCD3 bispecific antibodies against diffuse large B‐cell lymphoma (DLBCL) cell lines with different levels of expression of CD20
Source: Br J Haematol. 2025 Mar 3;206(5):1350–4. doi: 10.1111/bjh.20033 (PMC12078854; doi:10.1111/bjh.20033)
Supplement: Supplementary file 1 — Data S1. [file BJH-206-1350-s002.docx]

Supplementary Methods

**Cell Lines and Primary samples:** Cell lines used in this study were Karpas 1718 (tMZL, CVCL_2539, ECACC),^1^ SU-DHL-10 (GCB-DLBCL, CVCL_1889, DSMZ, ACC-576),^2^ UoL-AME and UoL-RAD, the latter both derived in Leicester from patients with primary immunochemotherapy refractory DLBCL in leukaemic phase (SJ et al, manuscript in preparation). The Karpas 1718 cell line was established from a patient with transformed SMZL and SU-DHL-10 from a patient diagnosed with GCB-DLBCL, both derived from patients with no prior exposure to CD20 immunotherapeutics or CAR T-cells.^3,4^ All cell lines were authenticated by short tandem repeat profiling and routinely tested for mycoplasma contamination. Healthy volunteer blood sample collection was sponsored by the University of Leicester and approved by a research ethics committee (2024-0243-476).

**Protein expression analysis:** Cells were lysed in lysis buffer (0.5% NP40, 125mM NaCl, 50mM Tris-HCl, 1mM DTT, protease inhibitors) and protein concentration quantified using Biorad Protein Assay. Antibodies used were anti-CD20 (Thermo Fisher, 14-0202-37) and anti-α-tubulin (Thermo Fisher, 14-4502-82).

**QiFi Quantification of Surface CD20:** The QiFi kit (Agilent Dako, K007811-8) was used for quantification of cell surface CD20 expression of the cell lines according to manufacturer’s instructions. Cells were stained with a α-CD20 primary Ab (BD 555410) or isotype control (BD 555740) for 1 hour prior to staining with a α-mouse FITC secondary Ab. Cells were fixed 1% paraformaldehyde (PFA) resuspended in FACS buffer. The QiFi beads were washed and stained with secondary Ab as per the cell line samples.

**Assessment of CD20 Antibody Binding:** Cell lines (UoL-RAD, SU-DHL-10, Karpas 1718 and UoL-AME) were incubated with 10nM CD20 antibody biosimilar for 1 hour at 4^o^C. Cells were stained with LIVE/DEAD Fixable Green (Thermo Fisher, L23101) for 30 minutes, then incubated with anti-human IgG-APC (BioLegend, 366906) for 30 minutes. 10,000 live cell events were acquired on a CytoFLEX flow cytometer (Beckman Coulter).

**Cytotoxicity Assays:** Effector cells were derived from PBMCs prepared using standard density-gradient isolation followed by B-cell depletion with CD20 Microbeads (Miltenyi 130-091-104). Target cells (UoL-RAD, SU-DHL-10, Karpas 1718 and UoL-AME) were pre-stained with CellTrace Yellow (Thermo Fisher, C34567). Effector:target cell ratio was 6:1. BsAb biosimilars epcoritamab (Proteogenix, PX-TA1587-100), glofitamab (Proteogenix, PX-TA1590-100), odronextamab (Proteogenix, PX-TA1599-100) and mosunetuzumab (Proteogenix, PX-TA1482-100) or CD20 monospecific antibody biosimilars rituximab (PX-TA1010) and obinutuzumab (PX-TA1172) were used at a concentration range of 0.01-100,000pM. CD107a-BV711 antibody (Biolegend, 328640) was added at a concentration of 2 µg/ml to each well at the beginning of the incubation. Assays were incubated for 24 hours at 37°C and 5% CO_2_ in a humidified incubator. Target cell death was measured using LIVE/DEAD Fixable Violet (Thermo Fisher, L34963), and cells were stained with a panel of fluorophore-conjugated antibodies - CD3-AF594 (Biolegend 300446), CD4-PerCP-Cy5.5 (Biolegend 300530), CD8-APC-Cy7 (Biolegend 301016), CD56-APC (Biolegend 318310), CD69-BV605 (Biolegend 310938) and CD25-PE-Cy7 (Biolegend 302612). Samples were acquired on an Attune NxT flow cytometer (Thermo Fisher) with an acquisition volume of 100µl. A compensation matrix was applied to all samples.

**Data analysis**: % B cell depletion (BCD) was calculated by normalising the absolute cell number of remaining live target cells (CellTrace Yellow/LD-, gated on single cells) to no drug controls. Flow cytometry files were analysed in FlowJo (v10.8.1). Statistics and plots were generated in R (v4.2.1) for cytotoxicity assays and GraphPad Prism (v9.4.0) for all other experiments. Dose-response curves and absolute EC50 values were generated using a 4-parameter log logistic regression model (*drc* package in R).

1. <https://www>.culturecollections.org.uk/nop/product/karpas-1718

2. <https://www.dsmz.de/collection/catalogue/details/culture/ACC-576>

3. Martinez-Climent JA, Sanchez-Izquierdo D, Sarsotti E, Blesa D, Benet I, Climent J, Vizcarra E, Marugan I, Terol MJ, Sole F, Cigudosad JC, Siebert R, Dyer MJ, García-Conde J. Genomic abnormalities acquired in the blastic transformation of splenic marginal zone B-cell lymphoma. Leuk Lymphoma. 2003; 44(3): 459-64.

4. Epstein AL, Levy R, Kim H, Henle W, Henle G, Kaplan HS. Biology of the human malignant lymphomas. IV. Functional characterization of ten diffuse histiocytic lymphoma cell lines. Cancer. 1978; 42(5): 2379-91.
