# Supplementary material for: In vitro comparison of CD20xCD3 bispecific antibodies against diffuse large B‐cell lymphoma (DLBCL) cell lines with different levels of expression of CD20
Source: Br J Haematol. 2025 Mar 3;206(5):1350–4. doi: 10.1111/bjh.20033 (PMC12078854; doi:10.1111/bjh.20033)
Supplement: Supplementary file 2 — Table S1. [file BJH-206-1350-s001.docx]

**Table 1**: **Characteristics of CD20XCD3 BsAb and CD20 antibodies**

|  | Antibody structure | Format | CD3 clone | CD20 clone | Clinical data/approvals in DLBCL |
| --- | --- | --- | --- | --- | --- |
| Mosunetuzumab | 1:1 | IgG1 | CD3δε | 2H7 (type 1 epitope, shared by rituximab) | Not approved |
| Epcoritamab | 1:1 | IgG1 | CD3ε | 7D8 (type 1 epitope, shared by ofatumumab) | FDA and EMA approval |
| Glofitamab | 2:1 | IgG1 | CD3ε | By-L1 (type 2 epitope, shared by obinutuzumab) | FDA and EMA approval |
| Odronextamab | 1:1 | IgG4 | CD3δε | t3B9-10 (type 1 epitope, shared by ofatumumab) | EMA approval |
| Obinutuzumab |  | IgG1 |  | By-L1 | No monotherapy approvals |
| Rituximab |  | IgG1 |  | 2H7 | FDA and EMA approval |
